# Clinical Implementation of Chromosomal Microarray Analysis: Summary of 2513 Postnatal Cases

**DOI:** 10.1371/journal.pone.0000327

**Published:** 2007-03-28

**Authors:** Xinyan Lu, Chad A. Shaw, Ankita Patel, Jiangzhen Li, M. Lance Cooper, William R. Wells, Cathy M. Sullivan, Trilochan Sahoo, Svetlana A. Yatsenko, Carlos A. Bacino, Pawel Stankiewicz, Zhishu Ou, A. Craig Chinault, Arthur L. Beaudet, James R. Lupski, Sau W. Cheung, Patricia A. Ward

**Affiliations:** Department of Molecular and Human Genetics, Baylor College of Medicine, Houston, Texas, United States of America; Johns Hopkins Medical School, United States of America

## Abstract

**Background:**

Array Comparative Genomic Hybridization (a-CGH) is a powerful molecular cytogenetic tool to detect genomic imbalances and study disease mechanism and pathogenesis. We report our experience with the clinical implementation of this high resolution human genome analysis, referred to as Chromosomal Microarray Analysis (CMA).

**Methods and Findings:**

CMA was performed clinically on 2513 postnatal samples from patients referred with a variety of clinical phenotypes. The initial 775 samples were studied using CMA array version 4 and the remaining 1738 samples were analyzed with CMA version 5 containing expanded genomic coverage. Overall, CMA identified clinically relevant genomic imbalances in 8.5% of patients: 7.6% using V4 and 8.9% using V5. Among 117 cases referred for additional investigation of a known cytogenetically detectable rearrangement, CMA identified the majority (92.5%) of the genomic imbalances. Importantly, abnormal CMA findings were observed in 5.2% of patients (98/1872) with normal karyotypes/FISH results, and V5, with expanded genomic coverage, enabled a higher detection rate in this category than V4. For cases without cytogenetic results available, 8.0% (42/524) abnormal CMA results were detected; again, V5 demonstrated an increased ability to detect abnormality. Improved diagnostic potential of CMA is illustrated by 90 cases identified with 51 cryptic microdeletions and 39 predicted apparent reciprocal microduplications in 13 specific chromosomal regions associated with 11 known genomic disorders. In addition, CMA identified copy number variations (CNVs) of uncertain significance in 262 probands; however, parental studies usually facilitated clinical interpretation. Of these, 217 were interpreted as familial variants and 11 were determined to be *de novo*; the remaining 34 await parental studies to resolve the clinical significance.

**Conclusions:**

This large set of clinical results demonstrates the significantly improved sensitivity of CMA for the detection of clinically relevant genomic imbalances and highlights the need for comprehensive genetic counseling to facilitate accurate clinical correlation and interpretation.

## Introduction

Genomic imbalances are a major cause of congenital and developmental abnormalities including dysmorphic features, mental retardation and developmental delay, and multiple congenital anomalies. Using routine cytogenetics techniques, chromosomal imbalances such as aneuploidies and segmental aneusomies must be larger than 3–5 Mb in size to be detected by GTG-banding at 450–600 band resolution [1]. In the past two decades, traditional banding has been combined with targeted molecular technologies to improve the resolution at which one can detect genomic changes. Fluorescence In Situ Hybridization (FISH) [2], Comparative Genomic Hybridization (CGH) [3] and Multiplex-FISH [4] or Spectral Karyotyping (SKY) [5] have been used to detect additional complex or submicroscopic abnormalities; however, such technologies are not suitable for whole genome scans in routine clinical testing because they either lack the necessary resolution or are too time consuming, labor intensive, and/or costly [6]–[8]. Array CGH technology has higher resolution and excellent throughput when compared with conventional and molecular cytogenetics [9]–[11]. In array CGH, genomic DNA from the patient and reference are labeled with different fluorescent dyes and co-hybridized to the array matrix containing clone DNA. The content of an array may include specific targeted regions of the genome or the entire genome arrayed on a single glass slide. Similar to conventional CGH, genome imbalances are quantified by analyzing the ratio of the two fluorescent hybridizing signals. The resolution of array CGH is determined by the size and number of the clones placed on the array to interrogate genome copy number changes. Using 1-Mb average coverage (3500 clones) BAC/PAC array analysis was shown to detect cryptic chromosomal imbalances in patients with idiopathic mental retardation and multiple congenital anomalies [12]–[14]. A tiling path BAC array (32K clones) has been developed and implemented in the clinical research field to identify novel genes associated with disease [15]. Chromosomal specific arrays, such as those for distal chromosomes 1p, proximal 17p and chromosome 22, have been utilized to identify subtle deletions and map specific rearrangement breakpoints [16]–[18]. Using a combination of a 1 Mb interval array and a specific chromosome 8 array, Visser and colleagues were able to identify the critical genomic region and disease gene responsible for CHARGE syndrome [19]. Exon array CGH has also been reported as a method to measure copy-number changes of individual exons in selected genes [20], although its implementation to broad genome coverage and the ability to discriminate multi-copy-exon encoding domains at the whole genome level remains to be determined. High-resolution array CGH has also been implemented in cancer research. For example, array-based methylated CpG island amplification (BAMCA) was developed to screen aberrant methylated CpG-rich sequences genome-wide in order to study the silencing of tumor suppressor genes [21].

Array CGH has unequivocally established its reliability in detecting copy-number changes, and several groups have now implemented this technology in clinical evaluation of both postnatal [22]–[25] and prenatal patient samples [26]–[31]. In comparison with research arrays, the clinical arrays must be carefully designed to precisely detect genomic imbalances for which clinical interpretations can be rendered. Our group initially developed and validated a BAC/PAC array in our clinical cytogenetics laboratory for Chromosomal Microarray Analysis (CMA V4) [24]. This version contained 366 FISH verified BAC/PAC clones that span genomic regions implicated in over 43 known genomic disorders [32] as well as all 41 subtelomeric regions. Subsequently, an expanded version of the array, CMA V5 that contains 853 FISH verified clones was implemented that detects gains and losses due to genomic rearrangements associated with over 70 disorders. Expansion of genome coverage in clinical arrays is always accompanied by the detection of copy number variation (CNVs) that might represent benign polymorphic changes.

Here, we present clinical experience with two versions of CMA arrays in the evaluation of 2513 clinical postnatal patient samples studied in our clinical diagnostic laboratory between February 2004 and March 2006 (Table 1). We compare these two versions with respect to detection rates for pathological chromosomal abnormalities and for CNVs, the latter most often representing benign variation. Our substantive dataset enables robust assessment of the implementation of this technology in patient management and as an aid to the establishment of a specific etiologic diagnosis.

**Table 1:** General comparison of CMA results correlated with routine cytogenetic results

	CMA V4			CMA V5		
	Total cases	Abnormal cases	Detection rate (%)	Total cases	Abnormal cases	Detection rate (%)
**Total patients studied**	**775**	**59**	**7.6**	**1738**	**154**	**8.9**
**Patients with abnormal karyotype/FISH**	**44**	**28**	**63.6**	**73**	**45**	**65.7**
Karyotype/FISH performed prior to CMA	26	12		43	26	
Karyotype/FISH performed concurrently with CMA	18	16		30	19	
**Patients with normal karyotype/FISH**	**680**	**28**	**4.1**	**1192**	**70**	**5.9**
Karyotype/FISH performed prior to CMA	462	24		855	56	
Karyotype/FISH performed concurrently with CMA	218	4		337	14	
**Patients with karyotype/FISH not available**	**51**	**3**	**5.9**	**473**	**39**	**8.2**
Karyotype/FISH performed prior to CMA	27	2		97	10	
Karyotype/FISH performed concurrently with CMA	24	1		138	8	
Karyotype/FISH not ordered	0	0		238	21	

## Results

All clinically relevant abnormal CMA results including *de novo* single clone changes [33] described in this section are listed in the Supplementary Tables S1 and S2; case ID numbers V4-1∼59 were performed on CMA V4 and ID numbers V5-1∼154 were conducted on CMA V5.

### CMA Concordance with Abnormal Cytogenetics Findings

The molecular based and targeted CMA approach detected the majority (92.5%) of those genomic imbalances identified by cytogenetic studies, including numerical changes, deletions and duplications (N = 43) and two marker chromosomes (case V4-27 and case V5-115) as well as two cases with >50% mosaicism involving a ring chromosome 22 (case V4-50) or monosomy X (case V4-52). CMA further identified the additional material of unknown origin and delineated breakpoints on all derivative chromosomes, thereby often having the added advantage of refining genomic intervals involved in the imbalance (N = 26) such as in case V4-31, case V5-41 and case V5-35 (Table S1 and S2). Moreover, on CMA V4, (case V4-33) a microduplication at 17p11.2-p12, that includes both the Smith-Magenis syndrome (SMS) and Charcot-Marie Tooth disease type 1 A (CMT1A) critical regions, was observed in one patient previously reported as having an abnormal 47,XYY karyotype. The additional genomic imbalance detected by CMA may explain the clinical findings including growth retardation and dysmorphic features, which were inconsistent with 47,XYY karyotype in this patient. Likewise, in a patient (case V5-144) with autistic behavior, developmental delay and dysmorphic features for whom cytogenetic studies showed a 47,XXY karyotype, CMA V5 revealed a smaller gain in the pericentromeric region of chromosome 7 including the Williams-Beuren syndrome (WBS) critical region at 7q11.23, which was confirmed by FISH analysis to be caused by a mosaic small ring chromosome 7 in 33% of the cells.

As anticipated, neither CMA V4 nor V5 could detect the apparently balanced translocations (N = 12), inversions (N = 21), insertions (N = 2), fra(X) expansions (N = 2) or extremely low level (<10%) mosaicism (N = 3). CMA V4 also failed to identify one marker chromosome, but this could be due to the limited array coverage. However, CMA V5 identified gains of the DiGeorge syndrome/Velocardiofacial syndrome (DGS/VCFS) genomic region not identified in two patients undergoing cytogenetic evaluations, which described apparently balanced translocations. One patient (case V5-109) with developmental delay and ambiguous genitalia had a karyotype indicating 46,XX,t(2;9)(p25.3;p22.1) whereas the other patient (case V5-110) manifested developmental delay and dysmorphic features and the identified karyotype of a three-way translocation 46,XX,t(12;18;13)(q14;q21.3;q14.2). Thus, although CMA does not detect balanced translocations or inversions it may uncover other cryptic genomic imbalances that are not detected by conventional cytogenetics in patients with phenotypic abnormalities and apparently balanced rearrangements. Interestingly, V5 also detected an apparent low level mosaic trisomy 14 (case V5-143); although subsequently validated by karyotype and FISH, both of these methods identified only 2% of cells with trisomy 14 in PHA stimulated T-cells [34].

### Clinically Relevant Genomic Imbalances Identified by CMA in Cases with Normal Cytogenetics Findings

CMA identified genomic imbalances not detected by conventional clinical cytogenetic analyses in a total of 5.2% of the patients; this represents 4.1% (20 losses and 5 gains) and 5.9% (39 losses and 38 gains) rates using V4 or V5, respectively (Table 1). Each of these abnormalities was confirmed independently by repeated GTG-banding and/or FISH tests. The confirmatory method used was dependent on the size of the genomic imbalance detected by CMA. These findings indicate that increasing the genomic coverage on the V5 targeted array enabled higher resolution and a 44% higher rate of detection of genomic imbalance compared to V4.

All the patients studied in this category are summarized in 5 groups based on the clinical indications provided at time of referral in Table 2. In group I, which are the patients with DD/MR only, the CMA clinically relevant detection rate was 3.9%; in group II, which are patients whose clinical indication was a combination of DD/MR and DF or MCA, CMA detected clinically relevant imbalances in 5.6%. CMA was most clinically sensitive, with the highest detection rate at 8.4%, in Group III, which are the patients with dysmorphic features (DF), multiple congenital anomalies (MCA), or the combination of both. Our targeted CMA detected fewer (3.4%) genomic imbalances in the Group IV patients with autistic behaviors; however, this group of patients will be further analyzed using a higher density array. CMA sensitivity in group V patients whose clinical indications represent “others” (e.g., seizure disorders, learning disability, failure to thrive, etc.) was 5.3%.

**Table 2:** CMA results correlated with clinical indication in the patients with normal cytogenetic tests

Group	Clinical indication	Total NL cyto	Total ABNL CMA	CMA detection rate
I	DD/MR	620	24	3.9%
II	DD/MR+/−DF or MCA	375	21	5.6%
III	DF, MCA or both	299	25	8.4%
IV	Autism	146	5	3.4%
V	Others*	432	23	5.3%
	Total	1872	98	5.2%

DD: developmental delay; MR: Mental retardation; DF: Dysmorphic feature; MCA: Multiple congenital anomalies

* including seizure disorders, failure to thrive, short stature, speech delay, learning disability, etc.

NL: normal; ABNL: abnormal

There were three cases in the category reported to have normal karyotype, normal locus specific FISH test at the DGS/VCFS critical region and normal telomeric FISH test. CMA V4 identified a *de novo* gain in the Miller-Dieker lissencephaly syndrome (MDLS) critical region in 17p13.3 for one patient (case V4-35); V5 revealed one duplication at the DGS/VCFS located in 22q11.2 (case V5-103), and a *de novo* gain at 4q22.3 (case V5-31) that was detected by a single clone. CMA V5 also detected five cases with mosaicism for aneuploidies ranging from 10–60% (cases V5-121, V5-151∼V5-154) that were not detected by routine cytogenetic analysis. The somewhat surprising ability of CMA to identify mosaicism not detected by karyotype analysis may be due to the difference in samples used for each test, as only PHA-stimulated T-cells are analyzed in routine chromosomal analysis whereas CMA uses genomic DNA from whole blood containing multi-lineage nucleated cells [34].

### Clinically Relevant Genomic Imbalances Identified by CMA in Cases without a Karyotype

For this group of patients, the sensitivity for detecting clinically relevant genomic imbalances was 5.9% (3/51) on version 4 and 8.2% (39/473) on version 5, respectively (Table 1). All the genomic imbalances detected by CMA were verified using the most appropriate method, either GTG-banding or FISH. Using CMA V4, there were two cases identified with microdeletions, one at 1p36.3 (case V4-3) and one at the DGS/VCFS locus (case V4-51). Using CMA V5, 19 cases had interstitial microrearrangements (12 losses and 7 gains), and 14 cases were identified with subtelomeric rearrangements. In retrospective high-resolution GTG-banding analyses of these 42 abnormal cases, 24 of these genomic imbalances were not detectable by routine cytogenetic analysis (Table S1 and S2).

Notably, there was an increase in the overall detection rate of genomic imbalances with CMA V5 (8.9%) relative to CMA V4 (7.6%) (Table 1), indicating that the increase in coverage of genomic disorder regions in the array enables additional imbalances to be identified in these screening tests even in the absence of case selection on the basis of normal cytogenetic studies.

### Microdeletions and the Reciprocal Microduplications

**Figure 1:**
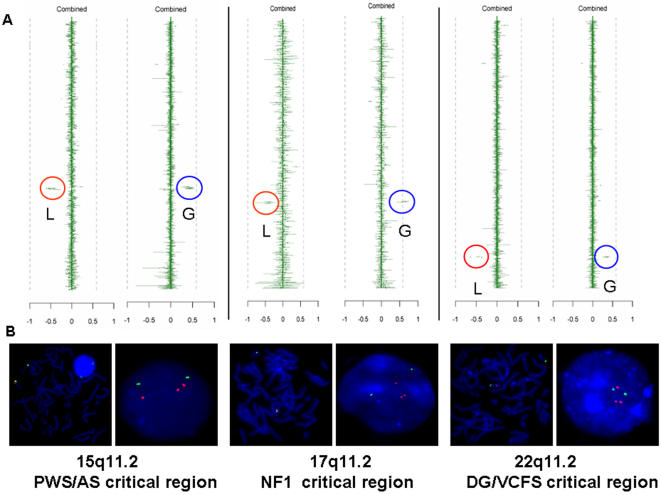
Example of reciprocal deletion/duplication CMA ratio plots with FISH validation. A Three sets of ratio plots showing examples of reciprocal microdeletion and microduplication at PWS/AS (on V5), NF1 (on V4) and DGS/VSFS (on V5) critical regions on chromosomes 15q11.2, 17q11.2 and 22q11.2, respectively. The vertical lines represent the BAC clones interrogating targeted regions of the genome, aligned according to their chromosomal position with the signal intensity ratios observed from the combined dye-swap experiments. Gains (G) are to the right and losses (L) to the left. Red circles highlight the signal ratios revealing genomic losses whereas the blue circles indicate copy number gains consistent with genomic deletions and reciprocal duplications, respectively. B FISH validation results. Control probes were labeled with a green chromophore and clones detecting deletions (in metaphase) or duplications (in interphase) were labeled in red for PWS/AS del/dup, NF1 del/dup and DG/VCFS del/dup.

There were 90 cases in which losses or gains associated with cryptic chromosomal microdeletions (51 cases) or the apparent predicted microduplications (39 cases) were detected (Table 3). Thirteen chromosomal regions associated with 11 known disorders involving microdeletions and microduplications, including 1p36 monosomy, 1q21.1 (novel), 1q44 (novel), Wolf-Hirschhorn syndrome (WHS) (4p16.3), cri-du-chat syndrome (CDCS) (5p15.3), WBS (7q11.23), WAGR (Wilm's tumor, aniridia, genital/or urinary tract abnormalities, mental retardation) (11p13), Prader-Willi/Angelman syndromes (PWS/AS) (15q11.2), MDLS (17p13.3), CMT1A/hereditary neuropathy with pressure palsies (HNPP) (17p12), SMS (17p11.2), neurofibromatosis type 1 (NF1) (17q11.2) and DGS/VCFS (22q11.2). For all 13 regions that were observed to undergo genomic losses, we also found cases with gains potentially representing reciprocal duplications; such events were observed at unequal frequencies (Table 3). The most commonly identified rearranged genomic regions include 1p36 (11 del/5 dup), 7q11.23 (6 del/3 dup), 15q11.2 (3 del/3 dup) and 22q11.2 (17 del/7 dup). Among these 90 cases, a subset of 70 patients (45 deletions and 25 duplications), had undergone previous cytogenetics and subtelomeric and/or locus specific FISH tests. Of these, 35 deletions (78%) and 23 duplications (92%) were not detected by conventional cytogenetic methods. Chromosomal regions at 1q21.1 (2 del/4 dup) and 1q44 (1 del/5 dup) include microrearrangements which have not been extensively reported in the literature. Genotype-phenotype correlation studies are ongoing to determine whether imbalances in these two regions represent novel genomic disorders [35]. Figure 1 illustrates examples of three pairs of microdeletions and apparent reciprocal duplication syndromes at 15q11.2q13, 17q11.2 and 22q11.2 detected by CMA V4 and V5.

**Table 3:** Microdeletions and the potential reciprocal duplications

Chromosome region	Deletion Syndrome	Duplication Syndrome	V4 Clone coverage	V5 Clone coverage	V4 del/dup	V5 del/dup	Total del/dup
1p36.3	1p36	dup 1p36	4	24	7/1	4/4	11/5
1q21.1	1q21.1*	dup 1q21.1	0	3	0/0	2/4	2/4
1q44	1q44*	dup 1q44	8	12	1/2	0/3	1/5
4p16.3	WHS	dup 4p16.3	8	8	0/1	4/0	4/1
5p15.3	CDCS	dup 5p15.3	3	6	0/0	2/4	2/4
7q11.23	WBS	dup 7q11.2	8	11	3/0	3/3	6/3
11p13	WAGR	dup 11p13	5	9	0/0	1/1	1/1
15q11.2	PWS/AS	dup 15q11.2q12	7	7	0/1	3/2	3/3
17p13.3	MDLS	dup 17p13.3	4	4	0/2	0/0	0/2
17p12	HNPP	CMT1A	3	3	0/1	1/1	1/2
17p11.2	SMS	Potocki-Lupski dup 17p11.2	4	4	0/1	1/0	1/1
17q11.2	NF1	dup 17q11.2	4	3	1/1	1/0	2/1
22q11.2	DGS/VCFS	dup 22q11.2	5	5	8/0	9/7	17/7
Total 90					20/10	31/29	51/39

* Chromosomal regions involved with microdeletions or microduplications that have not been reported previously.

Moreover, CMA V5 detected an additional eight cases with Xq28 gains that include the *MECP2* gene responsible for Rett syndrome [36]. Both CMA V4 and V5 illustrated improved potential over conventional cytogenetic analysis for identifying genomic imbalances associated with known microdeletions and evolving microduplication syndromes.

### Copy Number Variations (CNVs)

**Figure 2:**
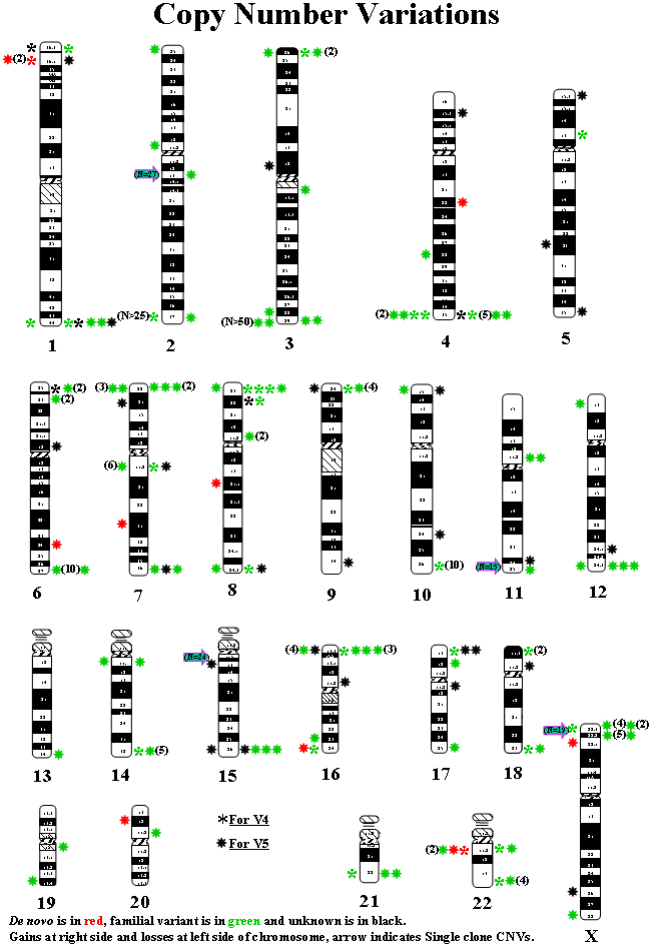
Ideogram of Copy Number Variations (CNVs). Big arrows indicate CNVs Type 1 (N = 119), except *AZFc* on the Y chromosome (N = 35). * represents V4 and for V5 for CNVs Type 2 (N = 143). A total of 262 copy number variations (CNVs) were identified by CMA. Of these, 217 were interpreted as familial variants (green) and 11 were determined to be *de novo* (red); 34 CNVs that await parental studies are depicted with unknown clinical significance (black). Positions showing losses are on the left side of the chromosome whereas gains are on the right side.

Two different types of apparent copy number variations were detected (Table 4). Multi-clone CNVs were classified as genomic segmental CNVs involving multiple interrogating clones from a single genetic locus whereas single clone CNVs represent genomic changes detected by only a single clone. Multi-clone CNVs, detected in 50 cases by CMA V4 and 69 cases on V5, were observed multiple times with either gains (in the majority of cases) or losses at five pericentromeric or subtelomeric regions including 2q13 (8/V4, 19/V5), 11q25 (14/V5), 15q11.2 (10/V4 and 14/V5), Xp22.3 (6/V4 and 13/V5), Yq11.2 (*AZFc*) (26/V4 and 9/V5) (Figure 2). Most of these changes were also found in the phenotypically normal parents, suggesting likely benign alterations; however, the potential clinical relevance of these CNVs remains unknown. We interpreted several multi-clone CNVs as likely representing normal variants by comparison to the Database of Genomic Variants [37] (http://www.sanger.ac.uk/PostGenomics/decipher).

**Table 4:** Copy Number Variations (CNVs)

	V4	V5	V4 detected	V5 detected
Multi-clone CNVs	50	69	6.5%	4%
Single clone CNVs	34	109	4.4%	6.3%
Familial variant	27	71	3.5%	4.1%
De novo	2	9	0.3%	0.5%
Unknown significance	5	29	0.6%	1.7%

There were 34 clones on version 4 and 109 clones on version 5 that were noted to reveal single clone gains or losses (Figure 2). The results for each single clone CNV were independently confirmed by FISH. A total of 98 CNVs (27 on V4 and 71 on V5) were inherited from normal parents as determined by either parental FISH or CMA testing and thus reported as familial variants. The remaining 45 changes (seven on V4 and 38 on V5) were queried on the public normal variation database (Toronto database http://projects.tcag.ca/variation) and the database on array CGH published by Iafrate et al [38], and none of these were reported as known normal variants. Of the 45 single clone CNVs, there were 11 (two on V4 and nine on V5) that revealed *de novo* (Figure 2) rearrangements in comparison to parental samples and thus, these are considered potentially causative for a phenotype [33]. It should be noted that paternity testing was not performed for these cases. For example, a single clone deletion (RP5-888M10) at 1p36.1 on CMA V4 was seen in two patients (cases V4-1∼2) whose clinical phenotypic appears distinct from the 1p36.3 deletion syndrome and repeated CMA of one patient on V5, with expanded coverage to 1p36.1 region, showed a more than 10 Mb deletion proximal to the typical 1p36.3 syndrome region. Another single clone deletion (RP11-12L9) on chromosome 7 that encodes the *FOXP2* gene located at 7q31.1 was detected in case V5-48. Parental *FISH* studies with this clone further indicated that the second copy of this clone was inserted into one chromosome 10 in the mother, uncovering a complex rearrangement in the child. This rearrangement was interpreted to be the cause of patient's phenotype of speech and language impairment [39]. Finally, 34 clones revealing gains or losses were considered as CNVs with unknown clinical significance and await familial studies for interpretation.

## Discussion

### CMA Increases Detection of Genomic Imbalances

Array CGH offers the potential for high throughput and high-resolution genome analysis and is a less labor intensive approach than other molecular cytogenetic analyses. In this study, we illustrate the value of array CGH in a large collection of clinical data (2513 cases). The targeted array enabled the detection of genomic imbalances, especially in the patients with previous or concurrent normal cytogenetic tests that were referred for DF, MCA or both (Table 2). As the targeted genomic coverage was increased, 17 additional abnormal cases were detected on the V5 array, including six cases associated with the newly assayed genomic regions, nine cases involving newly added pericentromeric regions and two cases with gains/losses of subtelomeric clones (data not shown), thus resulting in an enhanced overall detection rate. This greater genomic coverage on V5 also increased the rate at which single clone CNVs were identified. The heightened overall detection rate using V5 may also be due to factors such as technical improvements, improved experience in the data interpretation, and better selection of the clone coverage. For example, selected reduction of clones revealing known multi-clone CNVs led to a very significant reduction (57.5%) in the identification of these CNVs in patient samples using targeted V5 versus V4 arrays (Table 4).

Submicroscopic or subtelomeric chromosomal rearrangements have been found in about 6% of patients with otherwise unexplained mental retardation or multiple congenital anomalies [8], [40]. Array CGH has the capability of identifying such subtelomeric cryptic imbalances [41], [42]. In addition to the previously identified interstitial and subtelomeric imbalances, our CMA detected also all genomic gains and losses in patients with both normal karyotypes and FISH results. Because subtelomeric FISH analyses routinely include only one clone for each chromosome end, smaller and/or interstitial rearrangements can be missed. Similarly, microduplications are often missed by metaphase FISH because the duplicated signals are in close proximity to each other and cannot be distinguished on metaphase chromosomes; interphase FISH is required to resolve the duplicated signals and it is important to differentiate duplication from replication in a G2 phase nucleus [43]. Using CMA we identified four cases with gains at telomeric regions that were missed in previous subtelomeric FISH analyses. One of these patients had dysmorphic features, developmental delay and an autistic-like behavioral phenotype. Normal results were reported for routine G-banding, subtelomeric and probe specific FISH (at DGS/VCFS region) and fragile×testing. CMA identified a three-clone *de novo* gain due to duplication at the MDLS locus on 17p13.3 and these findings were confirmed by FISH. This latter case further illustrates the power of CMA as a rapid diagnostic technology capable of simultaneously interrogating multiple regions of the genome in a single test.

Array CGH also has an advantage in detecting microdeletions and the reciprocal microduplications at both interstitial and subtelomeric regions. A common mechanism for recurrent interstitial microrearrangements has been determined to result from nonallelic homologous recombination (NAHR) (intrachromosomal, interchromsomal or intrachromatid unequal crossing-over) between low-copy repeats (LCRs) [44], [45]. Such NAHR may produce two reciprocal products; a tandem (or direct) duplication and a deletion. The *de novo* events are predicted to occur at similar frequencies unless there is rearrangement specific selection or ascertainment bias, and, thus, it might be anticipated that both events would be seen in the population with equal frequency unless prenatal lethality, a milder phenotype with less likelihood to be studied or reduced penetrance occurs for the duplication or the deletion. Several reciprocal recombination disorders were delineated under this hypothesis, including duplications causing CMT1A and deletions causing HNPP [43], [46] at 17p12 and SMS deletion and Potocki-Lupski dup(17)(p11.2p11.2) syndrome [47] as well as rearrangements in 7q11.23, 15q11.2 and 22q11.2 [48]. In this study, CMA identified 90 cases that involved 13 different reciprocal microduplication-microdeletion rearrangements (Table 3). The most common cytogenetic regions involved include 1p36.3, 15q11-q13, and 22q11.2. Two of 13 regions were novel and patients with these reciprocal rearrangements are now being analyzed in depth.

### Balanced Translocations and ArrayCGH

CMA is limited to identifying genomic imbalances. Thus, balanced chromosomal translocations, inversions and insertions are not detected. Balanced translocations, in theory, should be benign unless the breakpoint disrupts a dosage sensitive gene or conveys a position effect. This is particularly important to consider in patients who have an abnormal phenotype, but the karyotype appears to represent a *de novo* balanced translocation. Several groups have focused on array CGH studies of patients with abnormal phenotypes and apparently balanced (primarily *de novo*) translocations based on routine cytogenetic analysis. Ciccone [49] et al reported that three out of four of such patients with apparently balanced translocations were actually unbalanced, and had complex rearrangements. They proposed that the array detected imbalances accounting for the mental retardation and other phenotypic abnormalities observed. Using a 1 Mb genome array, a cryptic 0.15–1.5 Mb deletion was detected at one of eight breakpoints of an apparently balanced three-way chromosomal translocation in a patient with behavior problems [50]. In another study, involving ten patients with *de novo* balanced translocations and abnormal phenotypes, a 1 Mb array detected genomic imbalances in six patients [51]. Thus, array CGH has the potential to detect imbalances in a considerable percentage of patients who have apparently balanced translocations based on routine cytogenetics, particularly in patients with abnormal phenotypes. For this application, tiling path rather than focused arrays are more appropriate.

In patients with a previous cytogenetic study, CMA did not detect additional gains or losses in 12 patients with apparently balanced translocations (4 *de novo*, 2 familial and 6 unknown). There are three possible explanations. First, there were neither genomic regulatory elements nor genes disrupted at breakpoint regions or if they were, the genes were not dosage sensitive. Second, the negative CMA results of balanced translocations could reflect the limitation of our current array with respect to clone coverage at these chromosomal breakpoints. Third, the unbalanced rearrangements at breakpoints were too small to detect using BAC/PAC array technology. Although we did not identify any genomic imbalances at the breakpoint regions in patients who had previously reported balanced translocations, we did discover microduplications at 22q11.2 in two patients that might account for their abnormal phenotypes. A recently developed array painting technology [52], [53] could potentially aid in detection and further resolution of complex chromosomal translocations that appear to be balanced by providing higher resolution genome and accurate breakpoint analyses. Likewise, extremely high density microarrays, such as oligonucleotide arrays [54] could be used to characterize apparently balanced translocations, inversions, genomic imprinting and with the inclusion of oligonucleotides interrogating single nucleotide polymorphisms (SNPs) also detect uniparental disomy (UPD) [55].

### Increasing Clone Coverage with Targeted Arrays Detected More Genomic Imbalances but also Increased the Detection of Benign Variants

Several recent studies have emphasized the extent of copy-number variations (CNVs) in the human genome. Iafrate et al. used a human 1 Mb BAC array with ∼3000 clones on a panel of 55 unrelated individuals and found more than 200 clones detecting CNVs, 24 of which were present in >10% of the individuals; there was an average of 12.4 CNVs per individual [38]. Sebat et al. using oligonucleotide arrays to study only 20 individuals described 221 copy number differences representing 76 unique CNVs; they found an average of 11 CNVs per individual and the average length of the CNVs was 465 kb [56]. Both studies found 60–70 entire genes within CNVs. One study using oligonucleotide arrays found a median of 30 (range 19–34) CNVs per index case with mental retardation [57]. More recently, Goidts and colleagues found copy number differences by inter-species array CGH analysis, and a total of 322 sites of large-scale interspecies differences which were identified by comparing human genomes with those of other primates. They suggested that human-specific duplications (i.e. 2q13) may predispose to chromosomal rearrangement [58]. By using full coverage BAC array CGH, Wilson et al. discovered that human DNA copy-number is often increased relative to chimpanzee and gorilla. Several chromosomal regions, such as 1p36.13, 1q21.1, 2q13, 4p16.3, 7q11.23, 9p12, 15q11.2, were established with higher copy number changes in human [59]. Most of these regions overlap with those involved with known human microdeletions and microduplication syndromes [45], [60].

In the current study, the enhanced resolution of CMA V5 improved the overall detection rate of chromosomal abnormalities significantly (Table 5), but also identified more single clone CNVs; however, the multi-clone CNV detection rate was reduced (Table 4) due to improved array design. We identified two major types of CNVs according to likely phenotypic effects. One group involving five chromosomal regions 2q13, 11q25, 15q11.2, Xp22.3 and Yq12 (*AZFc*) were hypothesized as benign variations because of their presence in a reportedly unaffected parent. This interpretation is consistent with the CNV database (Toronto database http://projects.tcag.ca/variation). However, a continuing genotype-phenotype correlation study is required to better understand any potential relevance of these CNVs to a particular phenotype, particularly when inherited in combination as distinct alleles from each parent [60], [61]. CNVs involving at least 34 BAC/PAC clones remain of uncertain clinical significance; the majority of these CNVs involve gain (N = 26) detected by clones located at the telomeric/subtelomeric and pericentromeric regions. It is uncertain at present what proportion of these variations are benign versus pathological, and whether they occur with increased frequency near telomeres [62]. *De novo* gains or losses are much more likely to be pathological [33] compared to those inherited from a normal parent, and one study using SNP arrays interpreted the clinical significance almost entirely according to whether variation was inherited or *de novo* [57]. However, variants could occasionally have variable penetrance with the same allele causing a phenotype in the child but not the parent. Studies of variants within extended families may be required to determine if certain CNVs have variable penetrance for phenotypic effects. Additional investigations of family members, computational analysis in combination with published databases and systematic analysis of genomic polymorphisms will provide further insight into the plasticity of the human genome, therefore helping to determine if these are normal variations, disease susceptibility variants, or may cause disease in association with other alleles [60].

**Table 5:** Comparison of targeted array CGH clinical implementation studies

	V4	V5	Shaffer et al (2006)
Genomic clone coverage	366	853	831
Genomic region coverage	84	154	126
Total patients studied	749	1695	1500
aCGH abnormality	128 (17.1%)	274 (16.2%)	134 (8.9%)
Clinical relevant genomic imbalances	46 (6.1%)	125 (7.4%)	84 (5.6%)
Unknown clinical significance	5 (0.6%)	9 (0.5%)	14 (0.9%)
CNVs	77 (9.9%)	140 (8.1%)	36 (2.4%)

Cases with known abnormal cytogenetic results on both V4 and V5 were excluded

### Does Genomic Sequence Determine Chromosome Folding?

There were 14 cases on V4 and 46 cases on V5 reported with a normal karyotype and/or FISH performed either prior to CMA or concurrently with CMA. After CMA revealed the abnormalities of deletions and/or duplications ranging from 0.5 Mb to over than 10 Mb, a high resolution (at average 650 banding) retrospective karyotype was applied (Tables S1 and S2). In general, on V4, six cases (<3 Mb) of 14 showed no evidence of cytogenetic abnormalities while the remaining eight cases (>5 Mb) were found to have abnormal chromosomes. On V5, 33 cases showed a negative retrospective karyotype whereas the remaining 13 cases revealed visible chromosomal abnormalities. The estimated smallest rearrangement visible by cytogenetic banding was a 2.7 Mb loss (in case V5-28), and the estimated largest size aberration missed by traditional cytogenetics was a 7.2 Mb duplication (in case V5-59). However, the size of deletion or duplication estimated by CMA might not truly reflect the exact size of the rearrangement due to the limitation of the current targeted array with lack of clone coverage at some of the chromosome regions. Our data from retrospective karyotyping raises an important question: why are some changes of ∼3 Mb cytogenetically visible whereas ∼7 Mb changes may not be seen cytogenetically? Does this relate to the mechanism of packaging of the genome into chromosomes and genomic sequences required for chromosome folding revealed by G-banding? Potential explanations for discrepancies between genome size and cytogenetic detection include: 1) altered trypsin access to the folded chromosomes, 2) chromosomes failing to stretch during the preparations in the region of genomic imbalances or, 3) the distribution of chromosome condensation and the hierarchical folding [63], [64]. Shopland and colleagues found that the gene distribution pattern in primary sequence contributes to chromosome folding and organization in mice [65], and thus genomic sequence could be involved in the determination of chromosome folding. Systematic evaluation of genomic sequences from regions discrepant for rearrangement size visualized by arrays versus G-banding may reveal further insight into chromosome folding.

### Future Perspectives

In conclusion, this study demonstrates that CMA is a valid methodology for clinical testing. CMA is equivalent to performing hundreds of subtelomeric and locus specific FISH tests simultaneously, and it also reliably detects duplications that are not usually seen by metaphase FISH. Thus, CMA offers an efficient and high-throughput alternative for detecting and characterizing genomic imbalances. An informed approach in targeted array CGH design can improve detection of clinically relevant genomic imbalances and reduce detection of common, apparently benign CNVs as shown for our V5.

We have designed a higher density array CMA V6 which will cover more disease specific regions, enhance the coverage of each chromosomal band and further incorporate interrogation of genomic regions with architectural features causing susceptibility to rearrangements [66]. By doing so, we should increase the detection of rearrangements associated with novel genomic disorders, and perhaps also increase the detection rate among patients with apparently *de novo* balanced translocations but having unexplained phenotypes as we showed for the V5 to V4 transition.

Much higher density arrays, such as the whole genome tiling path arrays [15] or oligonucleotide arrays [54], [66] are likely to be implemented in clinical diagnosis in the near future, but clinical interpretation and counseling will be challenging because of CNVs. A targeted oligoarray will eventually have the potential feasibility in design that enables detection of all clinically relevant genomic imbalances and, in combination with CNV databases, interpretation of benign polymorphic CNVs. Such high resolution genome analysis is a complementary alternative to DNA sequence based genetic testing and other biochemical and cytological evaluations for exploring the genetic etiologies of disease.

## Material and Methods

CMA was previously validated on DNA samples from human subjects under an IRB approved protocol. It was introduced in February 2004 as a clinical test coupled with FISH confirmation of potential imbalances ascertained after screening by CMA [24] (http://www.bcm.edu/cma/).

### Patients' Sample Collection

Peripheral blood samples from a total of 2513 patients were submitted to the BCM Clinical Cytogenetics Laboratory between February 2004 and March 2006 with a request for CMA. Most cases had either previous or concurrent conventional cytogenetic analysis by GTG-banding or FISH (Table 1). In general, patients referred for CMA analysis had variable clinical phenotypes, most often including: developmental delay and/or mental retardation (DD/MR), dysmorphic features (DF), multiple congenital anomalies (MCA), seizure disorders and autistic or other behavioral abnormalities. Clinical indications at the time of referral are summarized in 5 groups (Table 6). We also noted that for the patients in Group III, the majority (∼75%) of the patients were under age 2 at the time being tested, therefore, they were not fully evaluated for DD/MR.

**Table 6:** Summary of patient clinical indications at the time of referral

Group	Clinical indication	V4	V5	Total cases
I	DD/MR	272	566	838
II	DD/MR+/-DF or MCA	140	330	470
III	DF, MCA or both	119	255	374
IV	Autism	55	159	214
V	Others*	189	428	617
	Total	775	1738	2513

DD: developmental delay; MR: Mental retardation; DF: Dysmorphic feature; MCA: Multiple congenital anomalies

* including Seizure disorders, failure to thrive, short stature, speech delay, learning disabilities, etc.

Overall, 775 samples which were analyzed using CMA V4 and 1738 samples were studied using CMA V5 (Table 1).

There were a total of 117 patients (44 on V4 and 73 on V5) for whom cytogenetic studies were abnormal by GTG-banding analysis (N = 113) and FISH tests (N = 4). In 69 cases the cytogenetic studies were done previously whereas in 48 cases the cytogenetic and CMA studies were performed concurrently (Table 1). Unbalanced chromosomal abnormalities (N = 80) detected included: 18 numeric changes of whole chromosomes; 22 chromosomal deletions and five duplications; nine with additional chromosomal material of unknown origin; 17 with derivative chromosomes and three with marker chromosomes. There are six cases with mosaicism that included two ring chromosomes (90% and 6% mosaicism) and four numeric abnormalities (84% mosaic monosomy chromosome X, 8% mosaic trisomy chromosome X, 2% mosaic trisomy chromosome 14 and 1% mosaic trisomy chromosome 9). Apparently balanced chromosomal abnormalities subjected to further analysis (N = 37) were found to include 12 balanced translocations, 21 inversions, two insertions, and two fra(X) expansions (detected by molecular methods).

CMA studies were conducted on 1872 patients with a normal karyotype by GTG-banding, a subset of whom also had normal subtelomeric and/or locus specific FISH. CMA V4, which contained 366 arranged BAC/PAC clones, was used in 680 cases, in which 462 cases had cytogenetic tests prior to CMA and 218 cases were concurrent with CMA; CMA V5 with 853 interrogating clones was implemented in 1192 cases, there were 855 cases that had previous cytogenetic tests, whereas 337 cases whose cytogenetic tests were performed with CMA concurrently (Table 1).

There were 524 patients studied, 51 performed using CMA V4 and 473 using CMA V5, for whom no information was available regarding a previous or concurrent karyotype and/or FISH analysis. A subset of the cases on V5 was ordered for CMA only.

### Array CGH

The CMA array V4 consisted of 366 BAC/PAC clones and V5 consisted of 853 clones (Table S3). Both were designed to contain 3 to 10 BAC/PAC clones per genomic disorder specific locus and subtelomeric regions. The version CMA V5 covered over 70 genetic disorders, all the clinically relevant subtelomeric regions (expanded from 5 Mb to 10 Mb per region), pericentric clones for detecting marker chromosomes, and clones that broadly span chromosomes 13, 18, 21, X, and Y for detection of these most common aneuploidies and their mosaicism. Additional “backbone” interrogating clones along each chromosome were added to aid in the detection of chromosome imbalance outside of targeted regions.

Array technology for printing and hybridization for this study was described before [24] except for the additional use of a Tecan automated hybridization unit for some samples for CMA version 5 with slight modification of the manufacturer's recommended protocol (Tecan HS 4800 hybridization station, Tecan Group Ltd, Switzerland).

### Array Scan and Data Analysis

Arrays were scanned into 16-bit tiff image files using an Axon two-color microarray scanner (Model 4000B) and quantified using GenePix Pro 6.0 (Molecular Devices Corp, Union City, CA) or Bluefuse (BlueGnome, Cambridge, UK).

Data analysis was performed by a web-based software platform utilizing an analysis scheme in the R open source statistical computing language. The system generates results in the form of raw, normalized and integrated data for all clones analyzed from a single patient sample as described previously [24]. In addition to computing the log_2_ Test/Reference intensity ratio value, a single clone T-statistic and permutation based *p*-value is also computed thereby providing further criterion to determine whether a clone deviates significantly from the mean. In general, abnormal results can be classified as gain or loss. A gain is defined when the combined score of a clone is greater than 0.2 for the log Test/Reference ratios and T-statistics permutation based *p*-value is <0.05 while a loss is defined by the combined score being less than −0.2 with T-statistics permutation based p-value <0.05. If a gain or loss of a single or multiple clones are indicated, confirmatory FISH analyses are performed. There are several categories of results that were reported: (1) no abnormalities detected by this version of the microarray; (2) clinically significant abnormalities detected, which were known to be associated with a genetic condition and confirmed by either FISH analysis and/or chromosome analysis and/or partial karyotype; (3) copy number gain or loss of uncertain clinical significance. This latter category might involve either single clone or multiple clone copy number changes that were either (3a) confirmed by FISH analysis or (3b) NOT confirmed by FISH analysis. Subsequent parental FISH and/or CMA studies were requested to determine if the copy number changes represent familial variants or a *de novo* event and potentially a clinically significant finding.

### FISH Validation

Metaphase preparations from patient peripheral lymphocytes followed a standard protocol [46]. BAC or PAC clone DNA probes were labeled using Biotin-14-dUTP, Digoxigenin dUTP, Spectrum Orange/Green according to published protocols. FISH analysis was performed using the standard clinical cytogenetics laboratory protocol.

## Supporting Information

Table S1 Clinically Relevant Abnormal CMA Cases for Version 4
(0.14 MB DOC)

Table S2 Clinically Relevant Abnormal CMA Cases for Version 5
(0.34 MB DOC)

Table S3 Genomic Disorder loci coverage on CMA Version 4
(0.32 MB DOC)
