# Supplementary material for: Clinical Implementation of Chromosomal Microarray Analysis: Summary of 2513 Postnatal Cases
Source: PLoS One. 2007 Mar 28;2(3):e327. doi: 10.1371/journal.pone.0000327 (PMC1828620; doi:10.1371/journal.pone.0000327)
Supplement: Table S1 — Clinically Relevant Abnormal CMA Cases for Version 4 (0.14 MB DOC) [file pone.0000327.s001.doc]

**Supplementary Table 1. Clinically Relevant Abnormal CMA Cases** for Version 4 (V4)

| ID | GB | ES | RGB | Clinical  indication | Karyotype | CMA results |
| --- | --- | --- | --- | --- | --- | --- |
| V4-1 | C | 20 | + | DF,MCA | 46,XY | arr cgh 1p36.11(RP5-888M10)dnx1 |
| V4-2 | C |  |  | DD/MR,SS | 46,XY,del(1)(p36.11p36.22) | arr cgh 1p36.11(RP5-888M10)dnx1 |
| V4-3 | P |  |  | FTT | NA | arr cgh 1p36.23(RP11-58A11->RP11-185B14)x1 |
| V4-4 | P | 5 | + | DD/MR,MC | 46,XX | arr cgh 1p36.33(GS-62L8->RP11-547D24)x1 |
| V4-5 | C |  |  | DD/MR | 46,XX,del(1)(p36.3) | arr cgh 1p36.33(GS-62L8->RP11-671C15)x1 |
| V4-6 | P | 7.5 | + | MCA | 46,XX | arr cgh 1p36.33(GS-62L8->RP11-92O17)x1 |
| V4-7 | C |  |  | DF,MCA,  CLP | 46,XX,del(1)(p36.13p36.32) | arr cgh 1p36.33(RP11-319A11->RP11-185B14)x1 |
| V4-8 | P | 1 | - | DD/MR,DF | 46,XX | arr cgh 1p36.3(RP4-628J24,RP11-547D24)x1 |
| V4-9 | P |  |  | DF,FTT | 46,XX,add(1)(p36.33) | arr cgh 1p36.33(GS-62L8->RP11-671C15)x1,5q35(RP11-2I16,GS-240G13)x3 |
| V4-10 | C |  |  | DD/MR,DF,  MCA | 46,XX,del(1)(q42.3) | arr cgh 1q44(RP11-339I11->GS-160H23)x1 |
| V4-11 | P | 9.5 | + | DD/MR | 46,XY | arr cgh 1q44(RP11-339I11->GS-160H23)x3 |
| V4-12 | P | 23 | + | DD/MR,  MCA | 46,XY | arr cgh 1q44(RP5-1090A23->GS-160H23)x3 |
| V4-13 | C |  |  | DD/MR,DF | 46,XY,der(3)t(3;7)(p25,q36) | arr cgh 3p26(RP11-385A18->RP11-33J20)x1,7q36.3(RP5-1015O24->GS-3K23)x3 |
| V4-14 | C |  |  | DD/MR,  SPD | 46,XY,add(3)(p25) | arr cgh 3p26(RP11-385A18->RP11-33J20)x1,14q32(RP11-796G6->GS-820M16)x3 |
| V4-15 | C |  |  | DF,MCA,  SD | NA | arr cgh 3q29(RP11-17P4,RP11-1D19)x3 |
| V4-16 | P | 1.7 | - | DF | 46,XY | arr cgh 4p16.3(GS-36P21->RP5-860A13)x3 |
| V4-17 | P | 0.2/? | - | AS | 46,XY | arr cgh 4q35(RP11-354H17)x1,10p12p14(RP11-120C13->RP11-56H7)x1 |
| V4-18 | P |  |  | NA | 46,XY | arr cgh 6p21.1(RP1-244F24->RP1-166H4)x1 |
| V4-19 | P | 3 | - | DD/MR,MC | 46,XX | arr cgh 6q27(RP11-91O16->RP3-495K2)x1 |
| V4-20 | C |  |  | MCA,AS | 46,XX,del(7)(p12.3p15.1) | arr cgh 7p14.1(RP11-706L12->RP11-2J17)x1 |
| V4-21 | P |  |  | DD/MR | 46,XY | arr cgh 7q11.23(RP4-439N19->RP4-665P5)x1 |
| V4-22 | C |  |  | DD/MR,  SPD | 46,XY | arr cgh 7q11.23(RP4-439N19->RP4-665P5)x1 |
| V4-23 | P |  |  | DD/MR,DF | 46,XX | arr cgh 7q11.23(RP4-439N19->RP4-665P5)x1 |
| V4-24 | P |  |  | ABNCH | 46,XY,invdup(8)(p)(qter-p23::p23-p11.2::p23-pter) | arr cgh 8p11.2p22(RP11-520F7->RP11-262I23)x3,8p23.3(RP11-555E9->CTD-2629I16)x1 |
| V4-25 | P |  |  | DD/MR | 46,XY | arr cgh 8p23.3(RP11-555E9, RP11-82K8)x1 |
| V4-26 | P |  |  | DD/MR | 46,XY,add(8)(p23.1) | arr cgh 8p23.3(RP11-555E9->CTD-2629I16)x1,12p13.3p12.3(GS-496A11->RP11-407G6)x3 |
| V4-27 | P |  |  | ABNCH | 45,X/47,XY,+mar | arr cgh 8p23.3p22(RP11-555E9->RP11-520F7)x3,8q 24.3(RP11-149P24->GS-489D14)x1,Y(15 BACs)x0 |
| V4-28 | P |  |  | DD/MR | 47,XY,+der(9)(details N/A) | arr cgh 9p24.3p13(GS-43N6->RP11-327L3)x3 |
| V4-29 | P | 2/6 | + | DD/MR,DF,  MCA,AS | 46,XY | arr cgh 9q34.3(RP11-216L13->GS-112N13)x1,Xp22.3(RP11-483M24->RP11-143E20)x3 |
| V4-30 | P |  |  | DD/MR,SD | 46,XY | arr cgh 10pter(GS-306F7,RP11-486H9)x3 |
| V4-31 | C |  |  | DD/MR | 46,XX,der(22)t(12;22)(p10;q13.3)[27]/46,XX[3] | arr cgh 12p13.3(GS-496A11->RP11-407G6)x3,22q13.3(RP11-66M5->GS-99K24)x1 |
| V4-32 | P |  |  | DD/MR,SD | 46,XX | arr cgh 15q11.2(-5E9->RP11-345N11)x1,Xp22.3(RP11-1325A17->RP13-167H21)x3 |
| V4-33 | P |  |  | DD/MR,DF,  FTT | 47,XYY | arr cgh 17p11.2p12(RP11-626C5->RP11-344E13)x3,Xp22.3(RP11-1325A17->RP13-167H21)x3,Y(15 BACs)x3 |
| V4-34 | P |  |  | DF | 46,XY | arr cgh 17p13.3(RP5-59D14,-95H6)x3 |
| V4-35 | P | 5 | - | DD/MR,AS | 46,XX,var(15ps+) | arr cgh 17p13.3(RP5-59D14->RP11-810M2)x3 |
| V4-36 | P | 5 | - | DD/MR,  FTT,MC | 46,XX | arr cgh 17q11.2(RP11-848P1,RP11-142O6)x3 |
| V4-37 | P |  |  | DF | 46,XX | arr cgh 17q11.2(RP11-848P1->RP11-805L22)x1 |
| V4-38 | C |  |  | DF | 47,XX,+18 | arr cgh 18(17 BACs)x3 |
| V4-39 | C |  |  | MCA | 46,XX,del(18)(p11.22) | arr cgh 18pterp11.2(GS-52M11->RP11-411B10)x1 |
| V4-40 | P |  |  | ABNCH | 46,XX,idic(18)(q23)[47]/r(18)[3] | arr cgh 18p11.2(RP11-105C15->RP11-411B10)x3,18q23(RP11-357H3->GS-964M9)x1 |
| V4-41 | C | 6 | + | NA | 46,XX | arr cgh 18q22(RP11-27C7->GS-964M9)x1 |
| V4-42 | P |  |  | NA | 46,XY | arr cgh 22q11.2(RP11-36N5)dnx1 |
| V4-43 | P |  |  | MCA | 46,XY,+21pstk | arr cgh 22q11.2(-F5->RP11-316L10)x1 |
| V4-44 | C |  |  | DD/MR,DF,  VSD | 46,XY | arr cgh 22q11.2(-F5->RP11-316L10)x1 |
| V4-45 | P |  |  | SD | 46,XX | arr cgh 22q11.2(-F5->RP11-165F18)x1 |
| V4-46 | C |  |  | DF | 46,XX | arr cgh 22q11.2(-F5->RP11-165F18)x1 |
| V4-47 | C |  |  | DD/MR,DF | 46,XY,ish del(22)(q11.21)(-F5) | arr cgh 22q11.2(-F5->RP11-165F18)x1 |
| V4-48 | C |  |  |  | 46,XX,ish del(22)(q11.21)(-F5) | arr cgh 22q11.2(-F5->RP11-165F18)x1 |
| V4-49 | P |  |  | MCA | 46,XY | arr cgh 22q11.2(-F5->RP11-186O8)x1 |
| V4-50 | C |  |  | DD/MR,MC | 46,XX,r(22)(p11.2q13.3)[18]/46,XX[2] | arr cgh 22q13.3(RP11-66M5->GS-99K24)x1 |
| V4-51 | P |  |  | VSD | NA | arr cgh 22q11.2(-F5-> RP11-316L10)x1 |
| V4-52 | P |  |  | ABNCH | 45,X[27]/47,XX,+21[5] | arr cgh X(66 BACs)x1 |
| V4-53 | P |  |  | DD/MR,DF | 47,XXX | arr cgh X(66 BACs)x3 |
| V4-54 | C |  |  | NA | 46,XX | arr cgh Xp22.3(RP11-1325A17->RP13-167H21)x1 |
| V4-55 | P | 16 | + | DD/MR,DF | 46,XY | arr cgh Xp22.3(RP11-483M24->CTC-285I15)x2,Yq11(RP11-336F2->RP11-79J10)x0 |
| V4-56 | C |  |  | DD/MR,  CHD | 47,XYY | arr cgh Xp22.3(RP11-1325A17->RP11-28P6)x2,Y(15 BACs)x2 |
| V4-57 | P |  |  | DD/MR | 47,XYY | arr cgh Xp22.3(RP11-1325A17->RP11-28P6)x2,Y(15 BACs)x2 |
| V4-58 | P |  |  | DD/MR,DF | 47,XYY | arr cgh Xp22.3(RP11-1325A17->RP11-28P6)x2,Y(15 BACs)x2 |
| V4-59 | P |  |  | DF | 47,XYY | arr cgh Xp22.3(RP11-1325A17->RP11-28P6)x2,Y(15 BACs)x2 |

*C: concurrent; P: previously; NA: not available; GB: GTG-Banding; RGB: Retrospective GTG-Banding

ES: Estimated genomic imbalance at Megabase resolution detected by CMA; “+”: cytogenetic visible in RGB; “-”: cytogenetic not visible in RGB

LD: learning disability

FTT: failure to thrive

SS: short stature

CHD: congenital heart defect

SD: seizure disorder

CLP: cleft lip and palate

MC: microcephaly

SPD: speech delay

AS: autistic Spectrum

HT: hypotonia

VSD: ventricular septal defect

ABNCH: chromosomal abnormalities
