# Supplementary material for: Clinical Implementation of Chromosomal Microarray Analysis: Summary of 2513 Postnatal Cases
Source: PLoS One. 2007 Mar 28;2(3):e327. doi: 10.1371/journal.pone.0000327 (PMC1828620; doi:10.1371/journal.pone.0000327)
Supplement: Table S2 — Clinically Relevant Abnormal CMA Cases for Version 5 (0.34 MB DOC) [file pone.0000327.s002.doc]

**Supplementary Table 2. Clinically Relevant Abnormal CMA Cases** for Version 5 (V5)

| ID | GB | ES | RGB | Clinical Indication | Karyotype | CMA results |
| --- | --- | --- | --- | --- | --- | --- |
| V5-1 | P | 2 | - | DD/MR | 46,XX | arr cgh 1p36.33(GS-62L8->RP11-547D24)x1 |
| V5-2 | P |  |  | LD | 46,XY | arr cgh 1p36.33(RP11-584P2)x3,Xp22.3(RP11-769N24,RP11-594H22)x1 |
| V5-3 | NA |  |  | DD/MR, DF,FTT | NA | arr cgh 1p36.2(RP11-185B14)dnx1 |
| V5-4 | P |  |  | DD,MCA | NA | arr cgh 1p36.2(RP11-340B24->RP11-285P3)x1 |
| V5-5 | NA |  |  | SZ | NA | arr cgh 1p36.33(GS-62L8->RP11-547D24)x1 |
| V5-6 | P |  |  | DD/MD | 46,XX,del(5)(p15.3) | arr cgh 1p36.33(GS-62L8->RP11-22L13)x3,5p15.3(RP11-43F13->RP11-227M19)x1 |
| V5-7 | P | 2 | - | DD/MR | 46,XX | arr cgh 1p36.33(GS-62L8->RP11-547D24)x3 |
| V5-8 | C | 5 | - | FTT | 46,XX | arr cgh 1q21(RP11-337C18->RP11-102F23)x1 |
| V5-9 | NA |  |  | DF,FTT | NA | arr cgh 1q21(RP11-337C18->RP11-102F23)x1 |
| V5-10 | C | 5 | - |  | 46,XX | arr cgh 1q21(RP11-337C18->RP11-102F23)x3 |
| V5-11 | P | 5 | - | DF | 46,XX | arr cgh 1q21(RP11-337C18->RP11-102F23)x3 |
| V5-12 | NA |  |  | DD/MR, DF | NA | arr cgh 1q21(RP11-337C18->RP11-102F23)x3 |
| V5-13 | P | 5 | - | DD/MR,  DF,FTT | 46,XX | arr cgh 1q21(RP11-337C18->RP11-102F23)x3 |
| V5-14 | P |  |  | DD/MR, DF | 46,XX,add(1)(p36) | arr cgh 1q44(RP11-370K11->GS-160H23)x3 |
| V5-15 | P | 1/0.2 | - | DD/MR, DF | 46,XX | arr cgh 1q44(RP11-342L9,RP11-690C23)x3 |
| V5-16 | P |  |  | DD/MR | NA | arr cgh 1q44(RP11-439E19,RP11-690C23)x3 |
| V5-17 | P |  |  | SS | 46,XX | arr cgh 2p24.3(RP11-123E16,RP11-495A22)x1 |
| V5-18 | P |  |  | ABNCH | 46,XY,add(7)(p22) | arr cgh 2pterp21(GS-892G20->RP11-443B20)x3,7p22.3(RP11-90P13->RP13-580H13)x1 |
| V5-19 | P |  |  | DD/MR, DF | 46,XX,add(4)(p16)[43]/46,XX[3] | arr cgh 2pterp25.1(GS-892G20->RP11-217D23)x3,4pterp16.3(GS-36P21->RP11-1365D11)x1 |
| V5-20 | C |  |  | DD | NA | arr cgh 2q13(RP11-566O4->RP11-528G9)x1 |
| V5-21 | P |  |  | DF | 46,XY,del(4)(p16) | arr cgh 2q13(RP11-322F4->RP11-528G9)x3,4p16.3p16.1(GS-36P21->RP11-338K13)x1 |
| V5-22 | P |  |  | ABNCH | 46,XX,der(2) | arr cgh 2q13(RP11-566O4->RP11-528G9)x1,2q37.1(RP11-415N16)x1 |
| V5-23 | P | 3.9 | + | DD/MR | 46,XX | arr cgh 2q37.2(RP11-5O24->RP11-491D2)x1 |
| V5-24 | P |  |  |  | 46,XY,der(3)t(3;7)(p26;p22.3) | arr cgh 3p26.3(RP11-385A18->RP11-392M7)x1,7p22.3(RP11-90P13->RP11-6A1)x3 |
| V5-25 | P |  |  | CHD | NA | arr cgh 3p26.3(RP11-385A18->RP11-392M7)x1,7pterp22.3(RP11-90P13->RP11-6A1)x3 |
| V5-26 | C | 3/5 | + | NA | 46,XX | arr cgh 3q29(RP11-1D19->RP11-803P9)x3,4q35(RP11-203L17->GS-963K)x1 |
| V5-27 | P |  |  | MC | 46,XY | arr cgh 3q29(RP11-432D10,RP11-447L10)x1 |
| V5-28 | P | 2.7 | + | DD, DF,FTT | 46,XY | arr cgh 4p16.3(GS-36P21->RP11-478C1)x1 |
| V5-29 | P | 5.2 | + | DD/MR, DF,SD | 46,XX | arr cgh 4p16.3(GS-36P21->RP11-326O23)x1 |
| V5-30 | P | 2 | - | DD/MR, DF,FTT,SD | 46,XX | arr cgh 4p16.3(GS-36P21->RP11-262P20)x1 |
| V5-31 | P | 5.6 | - | DD/MR, DF | 46,XY | arr cgh 4q22.3(RP11-369I16)dnx3 |
| V5-32 | P |  |  | DF | 46,XX,del(4)(q33) | arr cgh 4q35.1qter(RP11-203L17->GS-963K6)x1 |
| V5-33 | P | 3.5 | + | MCA | 46,XY | arr cgh 4q35.2(RP11-203L17->RP11-354H17)x1 |
| V5-34 | C |  |  | DD/MR,SD | 46,XX,der(14)t(5;14)(p13.3;q32.3) | arr cgh 5p13.3(RP11-43F13->RP11-91M12)x3,14q32.3(RP11-47P23)x1 |
| V5-35 | P |  |  | DF | 46,XX,der(21)t(5;21)(p14.2;q22.13) | arr cgh 5p15.3(RP11-43F13->RP11-91M12)x3,21q22.13(RP11-35C4->GS-63H24)x1 |
| V5-36 | C | 1/0.5 | - | MCA | 46,XY | arr cgh 5p15.3(RP11-327L20)x3,18qter(GS-964M9,RP11-89N1)x3 |
| V5-37 | P | 23/11 | + | DD | 46,XY | arr cgh 5p15.3p14.2(RP11-43F13->RP11-91L13)x3,21q22.11(RP11-17O20->GS-63H24)x1 |
| V5-38 | P | 8.8 | + | DD/MR | 46,XX | arr cgh 5p15.3p15.2(RP11-43F13->RP11-91M12)x1 |
| V5-39 | NA |  |  | SD | NA | arr cgh 5q35(RP11-1006E8->RP11-2I16)x1 |
| V5-40 | C |  |  | DD/MR, DF,SS | 46,XY,dup(6)(q21.3q25.1) | arr cgh 6q24.1(RP3-468K18)dnx3 |
| V5-41 | C |  |  | ABNCH | 46,XX,add(9)(p22) | arr cgh 6p25p24(RP1-20B11->RP11-380L24)x3,6q27(RP3-495K2)x3,9p24.3p21.2(GS-43N6->RP11-399M15)x1 |
| V5-42 | C |  |  | DD,DF | NA | arr cgh 7p21(RP11-51L23->RP11-71F18)x1 |
| V5-43 | C |  |  | DD,FTT | NA | arr cgh 7q11.23(RP5-1177A1->RP4-665P5)x1 |
| V5-44 | C |  |  | SD,CLP | NA | arr cgh 7q11.23(RP5-1177A1->RP4-665P5)x3 |
| V5-45 | NA |  |  | DF | NA | arr cgh 7q11.23(RP5-1177A1->RP4-665P5)x1 |
| V5-46 | C |  |  | DD/MR, DF | 46,XY.ish del(7)(q11.23q11.23)(ELN) | arr cgh 7q11.23(RP5-1177A1->RP4-665P5)x1 |
| V5-47 | NA |  |  | SPD | NA | arr cgh 7q11.23(RP5-1177A1->RP11-229D13)x3 |
| V5-48 | P | 15 | + | DD | 46,XY | arr cgh 7q31.1(RP11-12L9)dnx1 |
| V5-49 | P |  |  | DD/MR, DF | 46,XX,der(8)t(8;10)(pter;qter) | arr cgh 8p23.3(RP11-555E9->RP11-16G12)x1,10q26.3(RP11-338O1->RP11-140A10)x3 |
| V5-50 | P |  |  | ABNCH | 46,XX,add(8)(p23.1) | arr cgh 8p23.3(RP11-555E9->RP11-16G12)x1,8p22(RP11-520F7,RP11-90I3)x3 |
| V5-51 | C | 5 | - | DF | 46,XY | arr cgh 8q12.2(RP11-33I11->RP11-174G1)x1 |
| V5-52 | C | 2 | - |  | 46,XX | arr cgh 8q24.3(RP11-149P24,RP11-610E3)x3 |
| V5-53 | P |  |  | AS | 46,XY | arr cgh 16p13.3(RP11-64L12,CTD-2517G10)x1 |
| V5-54 | P | 40/8 | + | FTT | 46,XX | arr cgh 9p24.3(GS-43N6->RP11-48M17)x3,15q26.1(RP11-437B10->RP11-89K11)x1 |
| V5-55 | P |  |  | MCA | 46,XY | arr cgh 9p24.3(GS-43N6,RP11-165F24)x3 |
| V5-56 | P |  |  | SS | 46,XX | arr cgh 9p24.3(RP11-165F24,RP11-31F19)x3 |
| V5-57 | P |  |  | NA | NA | arr cgh 9p24.3(RP11-165F24,RP11-31F19)x3 |
| V5-58 | P | 1.3 | - | DF,CP | 46,XY | arr cgh 9p24.3(GS-43N6->RP11-31F19)x3 |
| V5-59 | P | 5/7.2 | - | DD/MR, DF | 46,XY,22pstk++ | arr cgh 9p24.3(GS-43N6->RP11-509D8)x1,Xq27q28(RP11-37P24->RP4-671D9)x3 |
| V5-60 | C |  |  | DD/MR, DF | 46,XY,del(9)(q13q21.2) | arr cgh 9q13q21.1(RP11-764O9,RP11-89K20)x1 |
| V5-61 | P |  |  | ABNCH | 46,XX,der(9)( (pter->q22.33::q31.1->q22.2::q31.2->qter) | arr cgh 9q22.3(RP11-173G21->RP11-332M4)x3 |
| V5-62 | P | 5 | - | DF,FTT | 46,XX | arr cgh 9q34.3(RP11-447M12->GS-112N13)x1 |
| V5-63 | P |  |  | DD/MR | NA | arr cgh 10q26.2(RP11-422P15->RP11-140A10)x1 |
| V5-64 | P | 5 | + | DD/MR | 46,XX | arr cgh 10q26.3(RP11-264E18->RP11-140A10)x1 |
| V5-65 | NA |  |  | NA | NA | arr cgh 10q26.3(RP11-338O1,RP11-435D11)x3,10q26.3(RP11-25C24->RP11-140A10)x1 |
| V5-66 | P |  |  | NA | NA | arr cgh 10q26.3(RP11-338O1->RP11-140A10)x3 |
| V5-67 | NA |  |  | AS | NA | arr cgh 11p11.2(RP11-709C9,RP11-463K8)x3 |
| V5-68 | P | 1.7 | - | DF | 46,XX | arr cgh 11p13(RP11-702F20->RP1-74J1)x1 |
| V5-69 | C | 1.7 | - | DD/MR,SD | 46,XX | arr cgh 11p13(RP11-702F20->RP1-74J1)x3 |
| V5-70 | NA |  |  | DD/MR | NA | arr cgh 12p12(RP11-407G6,RP11-543P15)x1 |
| V5-71 | P | 5 | + | DD/MR | 46,XY | arr cgh 12p13.3(RP1-96H9,RP11-69M1)x3 |
| V5-72 | P |  |  | ABNCH | 46,XX,der(22)t(12;22)(p11.21;p12) | arr cgh 12pterp11.2(GS-496A11->RP11-8P13)x3 |
| V5-73 | NA | 32 Mos | - | DF | NA | arr cgh 12pterp11.2(GS-496A11->RP11-8P13)x3 |
| V5-74 | C |  |  | DD/MR | NA | arr cgh 13q33.3q34(RP11-313L9->RP11-569D9)x1 |
| V5-75 | P |  |  | DD/MR | ABNL 14q+ | arr cgh 14q32.3(RP11-164H13->RP11-47P23)x3 |
| V5-76 | C |  |  | DF | 46,XX,del(14)(q32.31)[16]/46,XX[4] | arr cgh 14q32.32(RP11-566J3->RP11-521B24)x1 |
| V5-77 | P |  |  | DD/MR,AS | 46,XX | arr cgh 15q11.2(RP11-484P15->RP11-345N11)x1 |
| V5-78 | C |  |  | DD/MR | NA | arr cgh 15q11.2q12(RP11-289D12->RP11-345N11)x3 |
| V5-79 | C |  |  | DD/MR | 46,XY,dup(15)(q11.2q13) | arr cgh 15q11.2q12(RP11-289D12->RP11-345N11)x3 |
| V5-80 | NA |  |  | DD/MR,SD | NA | arr cgh 15q11.2q12(RP11-484P15->RP11-345N11)x1 |
| V5-81 | C |  |  | DD/MR,SD | 46,XX | arr cgh 15q11.2q12(RP11-484P15->RP11-345N11)x1 |
| V5-82 | NA |  |  | DD/MR,FTT | NA | arr cgh 15q14(RP11-814P5,RP11-323I15)x3 |
| V5-83 | P | 3.3 | - | DD/MR | 46,XX | arr cgh 15q14(RP11-602M11,RP11-814P5)x1 |
| V5-84 | P |  |  | MCA | 46,XY | arr cgh 16p13.3(RP11-75P12,RP11-95J11)x3 |
| V5-85 | P | 6 | - | DD/MR,DF | 46,XX | arr cgh 16p13.3(GS-121I4->RP11-148F10)x3 |
| V5-86 | P | 2 | - | DD/MR,DF | 46,XY | arr cgh 16q24.3(RP11-21B21->GS-240G10)x3 |
| V5-87 | C |  |  | DD/MR,DF | 46,XY,del(17)(p11.2p11.2) | arr cgh 17p11.2(RP11-416I2->RP11-78O7)x1 |
| V5-88 | P |  |  | DD/MR | 46,XX | arr cgh 17p12(RP11-626C5->RP11-726O12)x1 |
| V5-89 | C | 2 | - | DD/MR | 46,XY | arr cgh 17p12(RP11-626C5->RP11-726O12)x3 |
| V5-90 | P |  |  | DD/MR,DF | 46,XX | arr cgh 17q11.2(RP11-848P1->RP11-805L22)x1 |
| V5-91 | C |  |  | DD/MR,DF | 46,XY,der(18)dup(18)(q22.1q22.3) del(18)(q23) | arr cgh 18q22(RP11-704G7->RP11-669I1)x3,18q23(RP11-357H3->RP11-89N1)x1 |
| V5-92 | P | 10 | + | DF,MCA | 46,XY | arr cgh 19q12q13.1(RP11-620G10->RP11-618P17)x1 |
| V5-93 | P | 2.5 | - | MCA | 46,XY | arr cgh 19q13.4(RP11-46I13,RP11-45K21)x3 |
| V5-94 | NA |  |  | SD | NA | arr cgh 20p13(RP11-530N10,GS-1061L1)x1 |
| V5-95 | P |  |  | DD/MR,DF | 46,XY | arr cgh 20p13(RP11-530N10->RP4-673D20)x1 |
| V5-96 | C |  |  | DD/MR,DF | 46,XY,der(21)t(20;21)(p12;q22.3) | arr cgh 20p13p12(RP11-530N10->RP11-49D9)x3 |
| V5-97 | NA |  |  | NA | NA | arr cgh 20q13.3(RP11-458E15->-204A16)x3 |
| V5-98 | C |  |  | CHD | 47,XY,+21 | arr cgh 21(15 BACs)x3 |
| V5-99 | C |  |  | ABNCH | 47,XY,+21 | arr cgh 21(15 BACs)x3 |
| V5-100 | P |  |  | ABNCH | 48,XY, +21,+mar[18]/47,XY,+21[2] | arr cgh 21(15 BACs)x3 |
| V5-101 | P |  |  | DF | 46,XX,del(21)(q22.1) | arr cgh 21q22.1(RP11-17O20->GS-63H24)x1 |
| V5-102 | C |  |  | SD | 46,XY,del(21)(q22) | arr cgh 21q22.3(RP11-35C4->GS-63H24)x1 |
| V5-103 | P |  |  | DD/MR,DF | 46,XX | arr cgh 22q11.2(RP11-186O8,RP11-316L10)x3 |
| V5-104 | P |  |  | ABNCH | 46,XY.ish del(22)(q11.2)(-F5,D22S75) | arr cgh 22q11.2(RP11-186O8->RP11-165F18)x1 |
| V5-105 | C |  |  | MCA | 46,XY,del(22)(q11.21q11.23) | arr cgh 22q11.2(RP11-186O8->RP11-165F18)x1 |
| V5-106 | P | 2.5 | - | NA | 46,XX | arr cgh 22q11.2(RP11-186O8->RP11-165F18)x1 |
| V5-107 | NA |  |  | NA | NA | arr cgh 22q11.2(RP11-186O8->RP11-165F18)x1 |
| V5-108 | C |  |  | FTT | NA | arr cgh 22q11.2(RP11-186O8->RP11-165F18)x1 |
| V5-109 | C |  |  | DD/MR | 46,XX,t(2;9)(p25.3;p22.1) | arr cgh 22q11.2(RP11-186O8->RP11-165F18)x3 |
| V5-110 | P |  |  | DD/MR,DF | 46,XX,t(12;18;13)(q14;q21.3;14.2) | arr cgh 22q11.2(RP11-186O8->RP11-165F18)x3 |
| V5-111 | P | 2.5 | - | DD/MR,DF | 46,XY | arr cgh 22q11.2(RP11-186O8->RP11-165F18)x3 |
| V5-112 | P | 1 | - | FTT | 46,XX | arr cgh 22q11.2(RP11-36N5)dnx1 |
| V5-113 | P |  |  | ABNCH | 47,XX,+idic(22)(q11.2) | arr cgh 22q11.2(RP11-91O6,RP11-319F4)x3 |
| V5-114 | C |  |  | DD/MR,  SPD | 45,XY,der(19)t(19;22)(q13.4q11.2),-22[18]/46,der(19)t(19;22)(q13.4q11.2),-22,inv dup(22)(q11.2)[2].ish del(22)(q11.2)(-F5)[15]/22q11.2(-F5x2)[5] | arr cgh 22q11.2(RP11-91O6->RP11-316L10)x1 |
| V5-115 | P |  |  | ABNCH | 47,XX,+mar | arr cgh 22q11.2(RP11-91O6->RP11-165F18)x3 |
| V5-116 | P |  |  | CHD | 46,XX,ish del(22)(q11.21)(-F5) | arr cgh 22q11.2(RP11-186O8->RP11-165F180)x1 |
| V5-117 | P | 2.5 | - | DD/MR | 46,XX | arr cgh 22q11.2(RP11-186O8->RP11-165F180)x1 |
| V5-118 | NA |  |  | DD/MR | NA | arr cgh 22q13.3(RP11-93F4->GS-99K24)x1 |
| V5-119 | P |  |  | DD/MR | NA | arr cgh 22q13.3(RP11-66M5->GS-99K24)x1 |
| V5-120 | C |  |  | DD,FTT,  SPD | NA | arr cgh 22q13.3(RP11-93F4->GS-99K24)x1 |
| V5-121 | P |  |  | NA | 46,XY | arr cgh mosaic X(66 BACs)x2 |
| V5-122 | C |  |  | DD/MR,DF | 47,XXY[65]/46,XY[5] | arr cgh X(66 BACs)x2 |
| V5-123 | P |  |  | DD/MR | 47,XXX | arr cgh X(66 BACs)x3 |
| V5-124 | NA |  |  | DD/MR | NA | arr cgh X(66 BACs)x2 |
| V5-125 | C |  |  | DD/MR,DF | 47,XXX | arr cgh X(66 BACs)x3 |
| V5-126 | NA |  |  | NA | NA | arr cgh X(66 BACs)x3 |
| V5-127 | P |  |  | DD,DF | NA | arr cgh Xp21.3p11.4(RP11-487M22->RP11-258I23)x3 |
| V5-128 | P |  |  | NA | NA | arr cgh Xp21.1(RP11-241G16->RP11-122N14)x0 |
| V5-129 | P |  |  | ABNCH | 45,X | arr cgh Xp22.3p21(RP11-1325A17->RP11-449L4)x0,Y(11BACs)x0 |
| V5-130 | C |  |  | DF | 46,X,der(Y)t(X;Y)(p22.13;p11.32) | arr cgh Xp22.3p22.2(RP11-449L4->-239F12)x2 |
| V5-131 | P |  |  | MC | NA | arr cgh X22.321.3(RP11-1325A17->RP11-126O22)x1 |
| V5-132 | P | 14.7 | - | MCA | 46,XY | arr cgh Xq26.3q28(RP1-137H15->RP11-157E12)x2 |
| V5-133 | P | 10 | + | DD/MR,DF,MC | 46,XX | arr cgh Xq27.3q28(RP11-51C14->RP11-164A8)x1 |
| V5-134 | P | 2.5 | - | DD/MR,DF | 46,XY | arr cgh Xq28(RP11-157E12->RP4-671D9)x2 |
| V5-135 | NA |  |  | DD/MR | NA | arr cgh Xq28(RP11-157E12->RP4-671D9)x2 |
| V5-136 | P | 0.8 | - | NA | 46,XY | arr cgh Xq28(RP11-244I10-> RP4-671D9)x2 |
| V5-137 | P | 2 | - | DD/MR,DF,AS | 46,XY | arr cgh Xq28(RP11-54I20->RP4-671D9)x2 |
| V5-138 | P |  |  | CP | 46,XY | arr cgh Xq28(RP11-54I20->RP4-671D9)x2,4p16.3(RP11-338K13)x3 |
| V5-139 | P | 0.8 | - | DD/MR,DF,AS | 46,XY | arr cgh Xq28( RP11-244I10->RP4-671D9)x2 |
| V5-140 | P |  |  | DD/MR,DF,SS | 47,XYY | arr cgh Y(15BACs)x2 |
| V5-141 | C |  |  | NA | 46,XY | arr cgh Yp11.2(RP11-115H13->RP11-418M8)x0 |
| V5-142 | C |  |  | NA | 46,XX | arr cgh15q26.3qter(RP11-308P12->RP11-89K11)x1,Xq28(RP11-244I10->RP4-671D9)x3 |
| V5-143 | P |  |  | HT | 47,XX,+14[2]/46,XX[98] | arr cgh mosaic 14(16 BACs)x3 |
| V5-144 | P |  |  | DD,DF,AS | 47,XXY | arr cgh X(66 BACs)x2,mosaic 7q11.23(RP5-1177A1->RP4-665P5)x3 |
| V5-145 | P |  |  | DD/MR | 46,XX | arr cgh 7p22.3(RO11-90P13)x3 |
| V5-146 | C |  |  | DF | 46,XY | arr cgh 20p12.3(RP11-116E13)dnx1 |
| V5-147 | P |  |  | FTT,SS | 46,XX | arr cgh 16q24.3(RP11-104N10)dnx1 |
| V5-148 | P | 5 | - | NA | 46,XY | arr cgh Xp21.2(RP11-662D2)dnx1 |
| V5-149 | P |  |  | DD/MR,DF | 46,XY | arr cgh 8q21.1(RP11-90B7)dnx1 |
| V5-150 | NA |  |  | DD/MR,DF | NA | arr cgh mosaic 7q11.23(RP5-1177A1->RP4-665P5)x1 |
| V5-151 | P |  |  | DD/MR | 46,XX | arr cgh mosaic 9(38 BACs)x3 |
| V5-152 | C |  |  | CHD | 46,XY | arr cgh mosaic 22(19 BACs)x3 |
| V5-153 | P |  |  | DF,MC,HT | 46,XX | arr cgh mosaic 8(39 BACs)x3 |
| V5-154 | P |  |  | DF | 46,XX | arr cgh mosaic 9(38 BACs)x3 |

*C: concurrent; P: previously; NA: not available; GB: GTG-Banding; RGB: Retrospective GTG-Banding

ES: Estimated genomic imbalance at Megabase resolution detected by CMA; +: cytogenetic visible in RGB; - : cytogenetic not visible in RGB

LD: learning disability

FTT: failure to thrive

SS: short stature

CHD: congenital heart defect

SD: seizure disorder

CLP: cleft lip and palate

MC: microcephaly

SPD: speech delay

AS: autistic Spectrum

HT: hypotonia

VSD: ventricular septal defect

ABNCH: chromosomal abnormalities
