# Supplementary material for: Clinical Implementation of Chromosomal Microarray Analysis: Summary of 2513 Postnatal Cases
Source: PLoS One. 2007 Mar 28;2(3):e327. doi: 10.1371/journal.pone.0000327 (PMC1828620; doi:10.1371/journal.pone.0000327)
Supplement: Table S3 — Genomic Disorder loci coverage on CMA Version 4 (0.32 MB DOC) [file pone.0000327.s003.doc]

**Supplementary Table 3. Genomic Disorder loci coverage on CMA Version 4 (V4) and Version 5 (V5)**

| OMIM# | Loci | Abnormalities detected  by CMA | Cytogenetic Abnormalities | Genes | Number of Clones on V 4 | Number of Clones on V 5 |
| --- | --- | --- | --- | --- | --- | --- |
|  | 1p36 | 1p36 deletion and/or duplication/triplication | 1p36 deletion and /or duplication |  | 8 | 24 |
|  |  | 1p pericentromeric region | 1p12 |  |  | 3 |
|  |  | 1q pericentromeric region | 1q21 |  |  | 3 |
|  |  | 1q subtelomeric region | 1q43-q44 |  | 8 | 12 |
|  |  | 2p subtelomeric region | 2p25 |  | 4 | 11 |
| 164280 |  | Feingold syndrome | 2p24.3 deletion | *MYCN* |  | 3 |
| 603714 | HPE2 | Holoprosencephaly 2 | 2p21 deletion | *SIX3* |  | 3 |
|  |  | 2p pericentromeric region | 2p11.2 |  |  | 2 |
|  |  | 2q pericentromeric region | 2q11.2 |  |  | 3 |
| 256100 | NPH1 | Nephronophthisis 1 | 2q13 homozygous deletion | *NPHP1* | 3 | 3 |
|  |  | 2q subtelomeric region | 2q37 |  | 4 | 16 |
|  |  | 3p subtelomeric region | 3p26 |  | 4 | 12 |
|  |  | 3p pericentromeric region | 3p11 |  |  | 3 |
|  |  | 3q pericentromeric region | 3q11.2 |  |  | 4 |
| 220200 | DWS | Dandy-Walker syndrome | 3q24 deletion | *ZIC1 ZIC4* |  | 2 |
|  |  | 3q subtelomeric region | 3q29 |  | 4 | 10 |
|  |  | 4p subtelomeric region | 4p16 |  | 2 | 10 |
| 194190 | WHS | Wolf-Hirschhorn syndrome | 4p16.3 deletion |  | 6 | 8 |
|  |  | 4p pericentromeric region | 4p12 |  |  | 3 |
|  |  | 4q pericentromeric region | 4q12 |  |  | 3 |
| 180500 | RIEG1 | Rieger syndrome | 4q25 deletion | *PITX2* | 3 | 2 |
|  |  | 4q subtelomeric region | 4q35 |  | 4 | 6 |
|  |  | 5p subtelomeric region | 5p15 |  | 1 | 5 |
| 123450 | CdCS | Cri-du-Chat syndrome | 5p15.2-p13.3 deletion |  | 5 | 6 |
| 122470 | CdLS | Cornelia de Lange syndrome | 5p13.2 deletion | *NIPBL* | 3 | 3 |
|  |  | 5p pericentromeric region | 5p12 |  |  | 3 |
|  |  | 5q pericentromeric region | 5q11.2 |  |  | 3 |
| 117550 | SOS | Sotos syndrome | 5q35 deletion | *NSD1* | 5 | 3 |
|  |  | 5q subtelomeric region | 5q35 |  | 2 | 7 |
|  |  | 6p subtelomeric region | 6p25 |  | 4 | 12 |
| 119600 | CCD | Cleidocranial dysplasia | 6p21.1 deletion | *RUNX2* |  | 3 |
|  |  | 6p pericentromeric region | 6p12 |  |  | 1 |
|  |  | 6q pericentromeric region | 6q11 |  |  | 3 |
|  |  | 6q subtelomeric region | 6q27 |  | 4 | 11 |
|  |  | 7p subtelomeric region | 7p22 |  | 4 | 13 |
| 101400 | SCS | Saethre-Chotzen syndrome | 7p21.1 deletion | *TWIST* |  | 3 |
| 175700 | GCPS | Greig cephalopolysyndactyly syndrome | 7p14.1 deletion | *GLI3* | 3 | 3 |
|  |  | 7p pericentromeric region | 7p11.2-p12 |  |  | 1 |
|  |  | 7q pericentromeric region | 7q11.21 |  |  | 2 |
| 194050 | WBS | Williams-Beuren syndrome | 7q11.23 deletion | *ELN, LIMK1* | 11 | 11 |
| 142945 | HPE3 | Holoprosencephaly 3 | 7q36.3 deletion | *SHH* | 3 | 3 |
|  |  | 7q subtelomeric region | 7q36 |  | 5 | 6 |
|  |  | 8p subtelomeric region | 8p23 |  | 3 | 5 |
|  |  | 8p22 deletion/duplication syndrome | 8p22-p23.1 duplication/deletion |  | 5 | 5 |
|  |  | 8p pericentromeric region | 8p11.23 |  |  | 3 |
|  |  | 8q pericentromeric region | 8q11.21 |  |  | 3 |
| 214800 |  | CHARGE syndrome | 8q12.2 deletion | *CHD7* |  | 3 |
| 150230 | LGS | Langer-Giedion syndrome | 8q23.3-q24.11 deletion | *TRPS1 and/or EXT1* | 3 | 6 |
| 190350 | TRPS1 | Trichorhinophalangeal syndrome,Type I | 8q23.3 deletion | *TRPS1* | 4 | 6 |
|  |  | 8q subtelomeric region | 8q24 |  | 4 | 8 |
|  |  | 9p subtelomeric region | 9p24 |  | 4 | 12 |
|  |  | 9p pericentromeric region | 9p12 |  |  | 3 |
|  |  | 9q pericentromeric region | 9q21 |  |  | 2 |
| 109400 | BCNS | Gorlin syndrome | 9q22.32 deletion | *PTCH* |  | 3 |
| 161200 | NPS | Nail-Patella syndrome | 9q33.3 deletion | *LMX1B* |  | 2 |
|  |  | 9q subtelomeric region | 9q34 |  | 4 | 12 |
|  |  | 10p subtelomeric region | 10p15 |  | 4 | 9 |
| 146255 | HDR | Hypoparathyroidism, sensorineural deafness, renal dysplasia | 10p14 deletion | *GATA3* | 3 | 3 |
| 601362 | DGS2 | DiGeorge syndrome 2 | 10p14 deletion |  | 2 | 3 |
|  |  | 10p pericentromeric region | 10p11.21 |  |  | 2 |
|  |  | 10q pericentromeric region | 10q11.21 |  |  | 1 |
| 608071 | SHFM3 | Split hand/split foot syndrome 3 | 10q24.3 duplication | *SHSF3* | 3 | 3 |
|  |  | 10q subtelomeric region | 10q26 |  | 4 | 13 |
|  |  | 11p subtelomeric region | 11p15 |  | 2 | 8 |
| 130650 | BWS | Beckwith-Wiedemann syndrome | 11p15.5 duplication/deletion | *IGF2, LIT1, p57, H19* | 2 | 2 |
| 106210 | AN2 | Aniridia type 2 | 11p13 deletion | *PAX6* | 3 | 6 |
| 607102 | WT1 | Wilm's tumor 1 gene | 11p13 deletion | *WT1* | 2 | 3 |
| 194072 | WAGR | Wilm's tumor-aniridia-genitourinary syndrome | 11p13 deletion | *WT1/PAX6* | 5 | 9 |
| 601224 | PSS | Potocki-Shaffer syndrome | 11p11.2 deletion | *ALX4 and/or EXT2* | 4 | 4 |
|  |  | 11p pericentromeric region | 11p11.12 |  |  | 4 |
|  |  | 11q pericentromeric region | 11q12.1 |  |  | 4 |
| 161015 |  | Leukodystrophy | 11q14.2-q14.3 deletion | *NDUFV1* |  | 3 |
| 147791 | JBS | Jacobsen syndrome | 11q24-q25 deletion |  | 1 | 4 |
|  |  | 11q subtelomeric region | 11q25 |  | 4 | 7 |
|  |  | 12p subtelomeric region | 12p13 |  | 4 | 12 |
|  |  | 12p pericentromeric region | 12p11.21 |  |  | 3 |
|  |  | 12q pericentromeric region | 12q12 |  |  | 3 |
| 163950 | NS1 | Noonan syndrome | 12q24.13 deletion | *PTPN11* |  | 2 |
|  |  | 12q subtelomeric region | 12q24 |  | 4 | 12 |
|  |  | 13q pericentromeric region | 13q12.11 |  |  | 2 |
| 180200 | RB1 | Retinoblastoma | 13q14.2 deletion | *RB1* | 2 | 2 |
| 603073 | HPE5 | Holoprosencephaly 5 | 13q32.3 deletion | *ZIC2* | 3 | 3 |
|  |  | 13q subtelomeric region | 13q34 |  | 4 | 10 |
|  |  | 14q pericentromeric region | 14q11.2 |  |  | 4 |
|  |  | 14q subtelomeric region | 14q32 |  | 6 | 9 |
|  |  | 15q pericentromeric region | 15q11.2 |  |  | 3 |
| 176270, 105830 | PWS/AS | Prader Willi syndrome/Angelman syndrome | 15q11.2-q13 deletion | *SNRPN/ UBE3A* | 7 | 7 |
| 209850 |  | Autism | 15q11.2-q13 duplication |  | 6 | 8 |
| 142340 | HCD | Diaphragmatic hernia | 15q26.1-q26.2 deletion |  |  | 7 |
|  |  | 15q subtelomeric region | 15q26 |  | 3 | 6 |
|  |  | 16p subtelomeric region | 16p13 |  | 3 | 11 |
| 600273, 191092 | PKDTS/ TS 2 | Polycystic kidney disease/tuberous sclerosis 2 | 16p13.3 deletion | *PKD1 and/or TSC2* | 3 | 3 |
| 180849 | RSTS | Rubinstein-Taybi syndrome | 16p13.3 deletion | *CREBBP* | 4 | 3 |
|  |  | 16p pericentromeric region | 16p11.2 |  |  | 2 |
|  |  | 16q pericentromeric region | 16q12.1 |  |  | 3 |
|  |  | 16q subtelomeric region | 16q24 |  | 4 | 14 |
|  |  | 17p subtelomeric region | 17p13.3 |  | 3 | 8 |
| 247200 | MDLS | Miller-Dieker lissencephaly syndrome | 17p13.3 deletion | *LIS1 and/or YWHAE* | 3 | 4 |
| 118220 | CMT1A | Charcot-Marie-Tooth disease type 1A | 17p12 duplication | *PMP22* | 4 | 4 |
| 162500 | HNPP | Hereditary neuropathy with liability to pressure palsies | 17p12 deletion | *PMP22* | 4 | 4 |
| 182290 | SMS | Smith-Magenis /dup(17)(p11.2p11.2) syndrome | 17p11.2 deletion or duplication | *RAI1* | 4 | 4 |
|  |  | 17p pericentromeric region | 17p11.2 |  |  | 1 |
|  |  | 17q pericentromeric region | 17q11.2 |  |  | 2 |
| 162200 | NF1 | Neurofibromatosis 1 | 17q11.2 deletion | *NF1* | 3 | 3 |
| 114290 | CMPD | Campomelic dysplasia | 17q24.3 deletion | *SOX9* | 2 | 3 |
|  |  | 17q subtelomeric region | 17q25 |  | 5 | 11 |
|  |  | 18p subtelomeric region | 18p11.32-p11.22 |  |  | 11 |
| 142946 | HPE4 | Holoprosencephaly 4 | 18p11.31 deletion | *TGIF* | 3 | 3 |
|  |  | 18p pericentromeric region | 18p11.21 |  | 2 | 3 |
|  |  | 18q pericentromeric region | 18q11 |  |  | 4 |
|  |  | 18q subtelomeric region | 18q23 |  | 6 | 12 |
|  |  | 19p subtelomeric region | 19p13 |  | 7 | 10 |
|  |  | 19p pericentromeric region | 19p12 |  |  | 2 |
|  |  | 19q pericentromeric region | 19q12 |  |  | 3 |
|  |  | 19q subtelomeric region | 19q13.4 |  | 7 | 10 |
|  |  | 20p subtelomeric region | 20p13-p12.2 |  | 3 | 8 |
| 118450 | AGS | Alagille syndrome | 20p12.2 deletion | *JAG1* | 2 | 2 |
|  |  | 20p pericentromeric region | 20p11.21 |  |  | 3 |
|  |  | 20q pericentromeric region | 20q11.21 |  |  | 3 |
|  |  | 20q subtelomeric region | 20q13 |  | 3 | 11 |
|  |  | 21q pericentromeric region | 21q11.2 |  |  | 3 |
| 190685 | DS | Down syndrome critical region | 21q22 duplication |  | 1 | 1 |
| 236100 | HPE1 | Holoprosencephaly 1 | 21q22.3 deletion | *TMEM1* |  | 2 |
|  |  | 21q subtelomeric region | 21q22 |  | 4 | 8 |
|  |  | 22q pericentromeric region | 22q11.21 |  |  | 1 |
| 115470 | CES | Cat eye syndrome | 22q11.2 inverted duplication |  | 1 | 2 |
| 192430 | DGS1/VCFS | DiGeorge syndrome 1/ Velocardiofacial syndrome | 22q11.2 deletion | *TBX1* | 5 | 5 |
| 188400 |  | dup(22)(q11.2q11.2) syndrome | 22q11.2 duplication |  | 5 | 5 |
| 607379 | NF2 | Neurofibromatosis 2 | 22q12.2 deletion | *NF2* |  | 1 |
|  |  | 22q subtelomeric region | 22q13 |  | 4 | 8 |
|  |  | Xp/Yp subtelomeric region | Xp22/Yp11.32 |  | 1 | 6 |
| 127300 | LWD | Leri-Weill dyschondrosteosis | Xp22.33/Yp11.32 | *SHOX* | 3 | 3 |
| 300495 | AUTSX2 | Autism, X-linked, susceptibility to, 2 | Xp22.32 deletion | *NLGN4* |  | 2 |
| 308100 | STS | Steroid sulfatase deficiency | Xp22.31 deletion | *STS* | 1 | 2 |
| 308700 | KMS | Kallmann syndrome 1 | Xp22.31 deletion | *KAL1* | 1 | 2 |
| 309801 | MLS | Micophthalmia with linear skin defects | Xp22.2 deletion |  | 3 | 3 |
| 300474 | GKD | Glycerol kinase deficiency | Xp22 deletion | *GK* | 3 | 3 |
| 300200 | AHC | Congenital adrenal hypoplasia/ sex reversal | Xp21.2 deletion/duplication | *NROB1* | 1 | 2 |
|  |  | Xp pericentromeric region | Xp11.21 |  |  | 3 |
|  |  | Xq pericentromeric region | Xq11.2 |  |  | 3 |
| 300300 | BTK | Bruton agammaglobulinemia tyrosine kinase | Xq22.1 deletion | *BTK* | 1 | 1 |
| 312080 | PMD | Pelizaeus-Merzbacher disease | Xq22 duplication | *PLP1* | 2 | 3 |
| 306955 | HTX | X-linked heterotaxy | Xq26.3 deletion | *ZIC3* |  | 3 |
| 300123 | MRGH | Mental retardation X-linked | Xq27.1 deletion or duplication | *SOX3* | 2 | 3 |
| 312750 | RTT | Rett syndrome | Xq28 deletion | *MECP2* | 2 | 3 |
|  |  | Yp subtelomeric region | Yp11.31 |  |  | 5 |
| 480000 | TDY | Testis-determining factor on Y | Yp11.31 deletion | *SRY* | 1 | 1 |
|  |  | Yp pericentromeric region | Yp11.2 |  |  | 3 |
|  |  | Yq pericentromeric region | Yq11.21 |  |  | 3 |
| 415000 | AZFa* | Azospermia factor a | Yq11 deletion |  | 2 | 2 |
|  | AZFb* | Azospermia factor b | Yq11 deletion |  | 2 | 2 |
|  | AZFc* | Azospermia factor c | Yq11.23 deletion |  | 2 | 2 |
|  |  | Xq/Yq subtelomeric region | Xq28/Yq12 |  | 1 | 9 |
